# Commentary: A Breathing-Based Meditation Intervention for Patients with Major Depressive Disorder Following Inadequate Response to Antidepressants: A Randomized Pilot Study

**DOI:** 10.3389/fmed.2017.00037

**Published:** 2017-04-06

**Authors:** Skye McKennon, Sarah Elizabeth Levitt, Grzegorz Bulaj

**Affiliations:** ^1^LS Skaggs Patient Wellness Center, Chief Wellness Office, University of Utah, Salt Lake City, UT, USA; ^2^A Better Way Wellness, Salt Lake City, UT, USA; ^3^Department of Nutrition and Integrative Physiology, College of Health, University of Utah, Salt Lake City, UT, USA; ^4^Wellness and Integrative Health Center, Huntsman Cancer Institute, University of Utah, Salt Lake City, UT, USA; ^5^Department of Medicinal Chemistry, College of Pharmacy, Skaggs Pharmacy Institute, University of Utah, Salt Lake City, UT, USA

**Keywords:** mindfulness, depression, drug-resistant, mobile health, digital health, yoga, telemedicine, self-care, disease self-management

Recently, the American College of Physicians published clinical practice guidelines recommending yoga and other non-pharmacological modalities as non-invasive therapies for patients with back pain (1). Whether for back pain, depressive symptoms, or other chronic disorders, can we imagine doctors prescribing “breathing” and “yoga” together with pharmacotherapies? The answer may be forthcoming. An article by Sharma et al. (2) on non-pharmacological treatment of drug-resistant depression is a welcoming addition to growing clinical research on adjunct therapies where pharmaceutical drugs may produce inadequate responses. Developing adjunct therapies that incorporate non-pharmacological and self-management practices offers means not only to improve therapy outcomes but also to maintain health-related quality of life after reaching remission. Since chronically ill patients, caregivers, and health-care providers embrace challenges of refractory medical conditions, medication non-adherence, and increasing treatment costs, clinical findings related to the drug-resistant depression described in Sharma et al. (2) have a broader impact on treatments of chronic diseases.

The Sharma et al. article (2) reports clinical efficacy of the breathing-based practice called the Sudarshan Kriya Yoga (SKY) in patients with major depressive disorder (MDD) who did not respond to antidepressant drugs. SKY is a breathing-based meditative technique of slow, medium, and fast rhythmic breathing cycles, and it does not require meditation experience. The 8-week SKY yoga treatment consisted of intense, 1-week training and 6-session SKY practice (3.5 h/day), followed by 7 weeks of at-home practice (20–25 min/day) and once-a-week 1.5 h sessions. In this controlled study, 25 patients who were taking antidepressants were randomized to either the SKY or waitlist control groups. The primary endpoint was measured using the Hamilton Depression Rating Scale (HDRS), and the secondary points were measured using the Beck Depression Inventory and the Beck Anxiety Inventory scores. Outcome measures were taken at least 1 week prior to the treatment (baseline), and at 1 and 2 months of the study. After 4 and 8 weeks of SKY treatment, significant improvements in depression symptoms were observed, as compared to no changes in the waitlist group. For the intent-to-treat sample, 46% of patients had >50% reduction of HDRS score from baseline.

This is not the first report on clinical efficacy of SKY for the treatment of depressive symptoms (3–5); however, it is the first report on its effectiveness in patients who do not respond to antidepressants. The health-related effects and therapeutic potential of SKY were reviewed (6), and yoga-based interventions for mental health are supported by clinical evidence (7). A recent randomized study showed that mindfulness-based cognitive therapy was effective in patients with drug-resistant depression (8), consistent with previous findings (9). Noteworthy, the 5-week Hatha yoga study did not indicate additional benefits for patients with MDD who were taking antidepressant drugs (10). Yoga is considered as safe if practiced properly, and with few possible adverse effects (11), supporting its potential as adjunctive intervention, which may improve tolerability, compliance, and efficacy of concurrent treatments. While yoga and psychotherapy may appear attractive adjunct therapies for depression, more longitudinal studies are needed to optimize their clinical benefits.

Physiological effects of breathing meditation and yoga indicate their complex pleiotropic nature, while allowing to glean insight into possible antidepressant mechanisms. Research on neuronal mechanism of meditation techniques, including yoga, shows engagement of specific brain structures (e.g., anterior cingulate cortex) involved in autonomic cognitive and emotional regulation (12–14). Three-month yoga intervention in patients with depression increased serum BDNF levels and decreased cortisol levels (15); this is in accord with yoga supporting stress regulation through the hypothalamic–pituitary–adrenal axis (16). Yoga and breathing exercises can modulate specific brain waves, including increase in the alpha wave activity (17). Effects of yoga correlates with the amount of practice (18), further emphasizing the importance of “dosing” and total duration of yoga treatment for specific chronic medical conditions, for example, for back pain (19–22).

There are two broader aspects of the work of Sharma et al. (2), namely: (1) growing number of clinical studies of diverse non-pharmacological modalities as adjunct treatments of CNS disorders and (2) developing non-pharmacological therapies as digital therapeutics using the “software-as-medical-device” strategy (Figure 1). With respect to the first aspect, nutrition and physical activity are promising adjunct therapies. Dietary and probiotic therapies can decrease depressive symptoms (23, 24), also in patients with MDD (25, 26). Folic acid was shown to be effective in refractory depression (27, 28). Both low- and high-intensity physical activity can improve response to drug therapy for depression (29–31). Lower sertraline doses may be required in patients using physical activity (32). There is also an increasing body of clinical evidence that ketogenic, modified Atkins and low-glycemic diets, or music, are beneficial for seizure control in patients with drug-resistant epilepsy (33–38). Mindfulness-based interventions are effective for substance use disorders (39).

**Figure 1:**
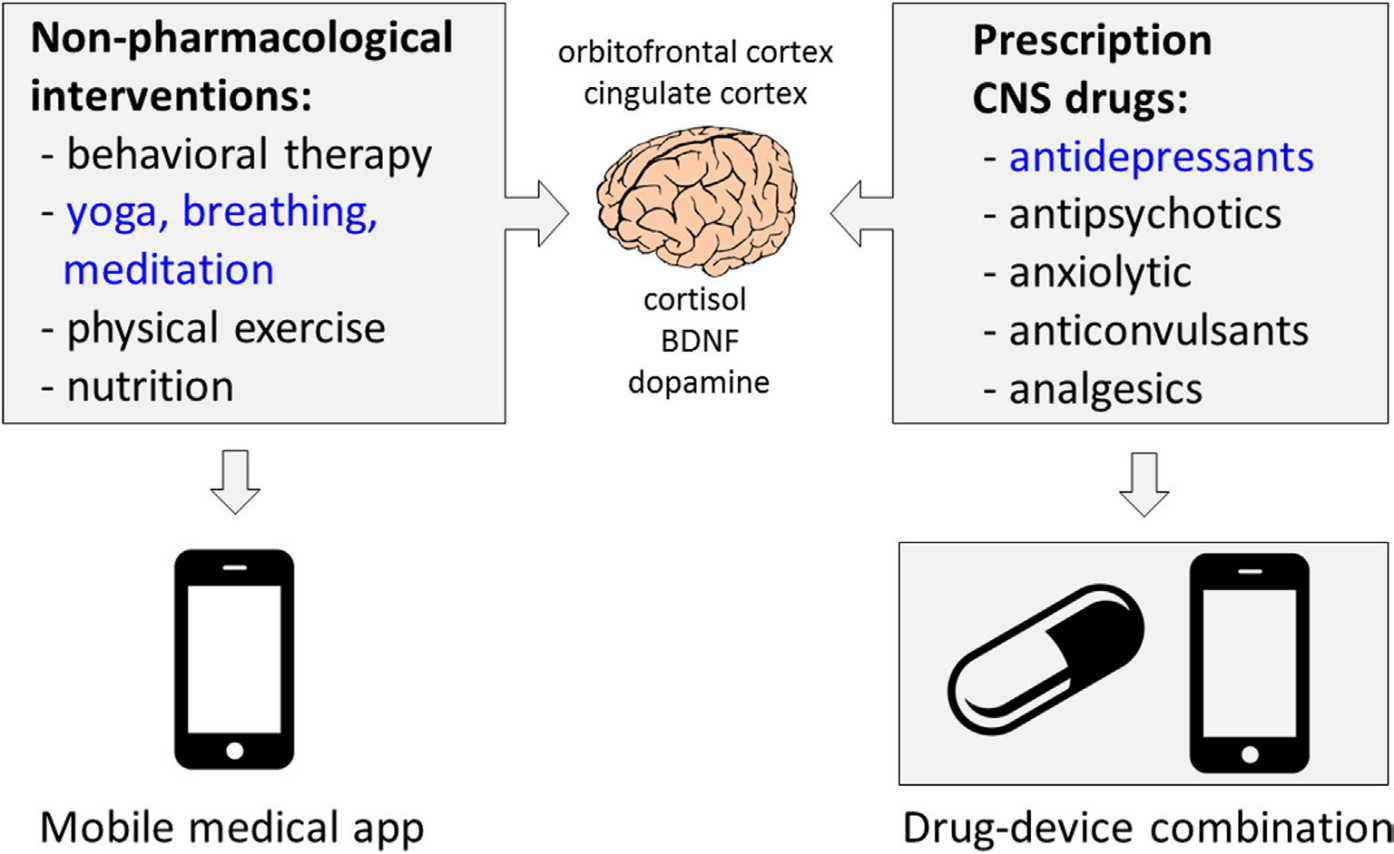
**Merging pharmacological and non-pharmacological interventions to improve patient engagement and therapy outcomes for depression and other CNS disorders**. Brain imaging and physiological studies suggest several overlapping mechanisms for CNS drugs and non-pharmacological interventions such as yoga, mindfulness, breathing meditations, and exercises (13, 40, 41). Delivering non-pharmacological interventions through telemedicine and medical mobile apps (software as medical device) opens long-term prospects to integrate disease-specific self-care with pharmacotherapies using drug–device combination products. Companies such as WellDoc, Pear Therapeutics, or Akili Interactive advance development of digital therapeutics for diverse chronic medical conditions. Regulatory agencies are involved in ensuring the patient’s safety and clinical efficacy of medical mobile apps (42).

With respect to the second broader aspect of the Sharma et al., mobile health (mHealth) technologies offer new opportunities for non-pharmacological modalities to be developed as adjunct therapies for the CNS disorders and delivered as drug–device combination products (43). There are several mobile apps and computer-based games that show clinical benefits in patients with depression (44–47). Given availability of mobile apps for deep breathing, yoga, nutrition, or physical exercise, it is feasible to integrate their contents in order to “repurpose” such mobile apps as digital interventions for people living with depression and other chronic disorders. Implementation of mHealth technologies includes pivotal clinical testing in order to receive regulatory approvals and to increase their acceptance among patients, primary care providers, and health-care industry, including third-party payers. A recent study suggested that mobile app intervention was more effective in people with moderate depression (44), emphasizing research needs for delineating specific clinical indications, in addition to improving clinical efficacy, patient engagement, and long-term effectiveness. This commentary aims to increase awareness of the medical research community and physicians about (1) clinical research on non-pharmacological adjunct therapies for depression and other CNS disorders and (2) opportunities to integrate non-pharmacological interventions with self-care using digital health technologies, including mobile software becoming medical devices.

## Author Contributions

SM, SL, and GB reviewed the literature and wrote the manuscript.

## Conflict of Interest Statement

GB is a cofounder and the officer of Epicadence PBC, Public Benefit Corporation, a company developing mobile software as medical device for epilepsy patients. GB is a coinventor of patented-technology “Disease Therapy Game Technology” and patent-pending “Multimodal Epilepsy Management Suite.” SM is a founder of A Better Way Wellness, a company promoting and coaching healthy lifestyles. SL declares no conflict of interest. The reviewer, JH, and handling editor declared their shared affiliation, and the handling editor states that the process nevertheless met the standards of a fair and objective review.

## References

[1] Qaseem A Wilt TJ McLean RM Forciea MA Clinical Guidelines Committee of the American College of Physicians. Noninvasive treatments for acute, subacute, and chronic low back pain: a clinical practice guideline from the American College of Physicians. Ann Intern Med (2017). 10.7326/M16-2367 28192789

[2] Sharma A Barrett MS Cucchiara AJ Gooneratne NS Thase ME. A breathing-based meditation intervention for patients with major depressive disorder following inadequate response to antidepressants: a randomized pilot study. J Clin Psychiatry (2017) 78(1):e59–63. 10.4088/JCP.16m10819 27898207 PMC5272872

[3] Doria S de Vuono A Sanlorenzo R Irtelli F Mencacci C. Anti-anxiety efficacy of Sudarshan Kriya Yoga in general anxiety disorder: a multicomponent, yoga based, breath intervention program for patients suffering from generalized anxiety disorder with or without comorbidities. J Affect Disord (2015) 184:310–7. 10.1016/j.jad.2015.06.011 26142611

[4] Janakiramaiah N Gangadhar BN Naga Venkatesha Murthy PJ Harish MG Subbakrishna DK Vedamurthachar A. Antidepressant efficacy of Sudarshan Kriya Yoga (SKY) in melancholia: a randomized comparison with electroconvulsive therapy (ECT) and imipramine. J Affect Disord (2000) 57(1–3):255–9. 10.1016/S0165-0327(99)00079-8 10708840

[5] Cramer H Anheyer D Lauche R Dobos G. A systematic review of yoga for major depressive disorder. J Affect Disord (2017) 213:70–7. 10.1016/j.jad.2017.02.006 28192737

[6] Zope SA Zope RA Sudarshan Kriya Yoga: breathing for health. Int J Yoga (2013) 6(1):4–10. 10.4103/0973-6131.105935 23440614 PMC3573542

[7] Balasubramaniam M Telles S Doraiswamy PM Yoga on our minds: a systematic review of yoga for neuropsychiatric disorders. Front Psychiatry (2012) 3:117 10.3389/fpsyt.2012.00117 23355825 PMC3555015

[8] Eisendrath SJ Gillung E Delucchi KL Segal ZV Nelson JC McInnes LA A randomized controlled trial of mindfulness-based cognitive therapy for treatment-resistant depression. Psychother Psychosom (2016) 85(2):99–110. 10.1159/000442260 26808973 PMC4756643

[9] Kenny MA Williams JM. Treatment-resistant depressed patients show a good response to Mindfulness-Based Cognitive Therapy. Behav Res Ther (2007) 45(3):617–25. 10.1016/j.brat.2006.04.008 16797486 PMC2808477

[10] Sarubin N Nothdurfter C Schule C Lieb M Uhr M Born C The influence of Hatha Yoga as an add-on treatment in major depression on hypothalamic-pituitary-adrenal-axis activity: a randomized trial. J Psychiatr Res (2014) 53:76–83. 10.1016/j.jpsychires.2014.02.022 24655586

[11] Cramer H Krucoff C Dobos G. Adverse events associated with yoga: a systematic review of published case reports and case series. PLoS One (2013) 8(10):e75515. 10.1371/journal.pone.0075515 24146758 PMC3797727

[12] Nakata H Sakamoto K Kakigi R. Meditation reduces pain-related neural activity in the anterior cingulate cortex, insula, secondary somatosensory cortex, and thalamus. Front Psychol (2014) 5:1489. 10.3389/fpsyg.2014.01489 25566158 PMC4267182

[13] Tang YY Holzel BK Posner MI. The neuroscience of mindfulness meditation. Nat Rev Neurosci (2015) 16(4):213–25. 10.1038/nrn3954 25783612

[14] Gard T Noggle JJ Park CL Vago DR Wilson A. Potential self-regulatory mechanisms of yoga for psychological health. Front Hum Neurosci (2014) 8:770. 10.3389/fnhum.2014.00770 25368562 PMC4179745

[15] Naveen GH Varambally S Thirthalli J Rao M Christopher R Gangadhar BN. Serum cortisol and BDNF in patients with major depression-effect of yoga. Int Rev Psychiatry (2016) 28(3):273–8. 10.1080/09540261.2016.1175419 27174729

[16] Gothe NP Keswani RK McAuley E. Yoga practice improves executive function by attenuating stress levels. Biol Psychol (2016) 121(Pt A):109–16. 10.1016/j.biopsycho.2016.10.010 27794449

[17] Desai R Tailor A Bhatt T. Effects of yoga on brain waves and structural activation: a review. Complement Ther Clin Pract (2015) 21(2):112–8. 10.1016/j.ctcp.2015.02.002 25824030

[18] Villemure C Ceko M Cotton VA Bushnell MC. Neuroprotective effects of yoga practice: age-, experience-, and frequency-dependent plasticity. Front Hum Neurosci (2015) 9:281. 10.3389/fnhum.2015.00281 26029093 PMC4428135

[19] Chang DG Holt JA Sklar M Groessl EJ. Yoga as a treatment for chronic low back pain: a systematic review of the literature. J Orthop Rheumatol (2016) 3(1):1–8. 10.13188/2334-2846.1000018 27231715 PMC4878447

[20] Nahin RL Boineau R Khalsa PS Stussman BJ Weber WJ. Evidence-based evaluation of complementary health approaches for pain management in the United States. Mayo Clin Proc (2016) 91(9):1292–306. 10.1016/j.mayocp.2016.06.007 27594189 PMC5032142

[21] Saper RB Boah AR Keosaian J Cerrada C Weinberg J Sherman KJ. Comparing once- versus twice-weekly yoga classes for chronic low back pain in predominantly low income minorities: a randomized dosing trial. Evid Based Complement Alternat Med (2013) 2013:658030. 10.1155/2013/658030 23878604 PMC3710634

[22] Wieland LS Skoetz N Pilkington K Vempati R D’Adamo CR Berman BM. Yoga treatment for chronic non-specific low back pain. Cochrane Database Syst Rev (2017) 1:CD010671. 10.1002/14651858.CD010671.pub2 28076926 PMC5294833

[23] Pirbaglou M Katz J de Souza RJ Stearns JC Motamed M Ritvo P. Probiotic supplementation can positively affect anxiety and depressive symptoms: a systematic review of randomized controlled trials. Nutr Res (2016) 36(9):889–98. 10.1016/j.nutres.2016.06.009 27632908

[24] Wallace CJ Milev R. The effects of probiotics on depressive symptoms in humans: a systematic review. Ann Gen Psychiatry (2017) 16:14. 10.1186/s12991-017-0138-2 28239408 PMC5319175

[25] Jacka FN O’Neil A Opie R Itsiopoulos C Cotton S Mohebbi M A randomised controlled trial of dietary improvement for adults with major depression (the ’SMILES’ trial). BMC Med (2017) 15(1):23. 10.1186/s12916-017-0791-y 28137247 PMC5282719

[26] Akkasheh G Kashani-Poor Z Tajabadi-Ebrahimi M Jafari P Akbari H Taghizadeh M Clinical and metabolic response to probiotic administration in patients with major depressive disorder: a randomized, double-blind, placebo-controlled trial. Nutrition (2016) 32(3):315–20. 10.1016/j.nut.2015.09.003 26706022

[27] Manosso LM Moretti M Rodrigues AL. Nutritional strategies for dealing with depression. Food Funct (2013) 4(12):1776–93. 10.1039/c3fo60246j 24154759

[28] Taylor MJ Carney SM Goodwin GM Geddes JR. Folate for depressive disorders: systematic review and meta-analysis of randomized controlled trials. J Psychopharmacol (2004) 18(2):251–6. 10.1177/0269881104042630 15260915

[29] Greer TL Trombello JM Rethorst CD Carmody TJ Jha MK Liao A Improvements in psychosocial functioning and health-related quality of life following exercise augmentation in patients with treatment response but nonremitted major depressive disorder: results from the tread study. Depress Anxiety (2016) 33(9):870–81. 10.1002/da.22521 27164293 PMC5662022

[30] Toups M Carmody T Greer T Rethorst C Grannemann B Trivedi MH. Exercise is an effective treatment for positive valence symptoms in major depression. J Affect Disord (2017) 209:188–94. 10.1016/j.jad.2016.08.058 27936452 PMC6036912

[31] Cooney GM Dwan K Greig CA Lawlor DA Rimer J Waugh FR Exercise for depression. Cochrane Database Syst Rev (2013) 9:CD004366 10.1002/14651858.cd004366.pub6 PMC9721454 24026850

[32] Siqueira CC Valiengo LL Carvalho AF Santos-Silva PR Missio G de Sousa RT Antidepressant efficacy of adjunctive aerobic activity and associated biomarkers in major depression: a 4-week, randomized, single-blind, controlled clinical trial. PLoS One (2016) 11(5):e0154195. 10.1371/journal.pone.0154195 27152523 PMC4859497

[33] Coppola G Toro A Operto FF Ferrarioli G Pisano S Viggiano A Mozart’s music in children with drug-refractory epileptic encephalopathies. Epilepsy Behav (2015) 50:18–22. 10.1016/j.yebeh.2015.05.038 26093514

[34] Felton EA Cervenka MC. Dietary therapy is the best option for refractory nonsurgical epilepsy. Epilepsia (2015) 56(9):1325–9. 10.1111/epi.13075 26198999

[35] Kim JA Yoon JR Lee EJ Lee JS Kim JT Kim HD Efficacy of the classic ketogenic and the modified Atkins diets in refractory childhood epilepsy. Epilepsia (2016) 57(1):51–8. 10.1111/epi.13256 26662710

[36] Lin LC Lee WT Wang CH Chen HL Wu HC Tsai CL Mozart K.448 acts as a potential add-on therapy in children with refractory epilepsy. Epilepsy Behav (2011) 20(3):490–3. 10.1016/j.yebeh.2010.12.044 21292560

[37] Dastgheib SS Layegh P Sadeghi R Foroughipur M Shoeibi A Gorji A. The effects of Mozart’s music on interictal activity in epileptic patients: systematic review and meta-analysis of the literature. Curr Neurol Neurosci Rep (2014) 14(1):420. 10.1007/s11910-013-0420-x 24272274

[38] Martin K Jackson CF Levy RG Cooper PN Ketogenic diet and other dietary treatments for epilepsy. Cochrane Database Syst Rev (2016) 2:CD001903 10.1002/14651858.CD001903.pub3 26859528

[39] Li W Howard MO Garland EL McGovern P Lazar M. Mindfulness treatment for substance misuse: a systematic review and meta-analysis. J Subst Abuse Treat (2017) 75:62–96. 10.1016/j.jsat.2017.01.008 28153483

[40] Mathersul DC Rosenbaum S. The roles of exercise and yoga in ameliorating depression as a risk factor for cognitive decline. Evid Based Complement Alternat Med (2016) 2016:4612953. 10.1155/2016/4612953 28044084 PMC5156813

[41] Muehsam D Lutgendorf S Mills PJ Rickhi B Chevalier G Bat N The embodied mind: a review on functional genomic and neurological correlates of mind-body therapies. Neurosci Biobehav Rev (2016) 73:165–81. 10.1016/j.neubiorev.2016.12.027 28017838

[42] Shuren J. The FDA’s role in the development of medical mobile applications. Clin Pharmacol Ther (2014) 95(5):485–8. 10.1038/clpt.2014.45 24747239

[43] Bulaj G. Combining non-pharmacological treatments with pharmacotherapies for neurological disorders: a unique interface of the brain, drug-device, and intellectual property. Front Neurol (2014) 5:126. 10.3389/fneur.2014.00126 25071711 PMC4095562

[44] Arean PA Hallgren KA Jordan JT Gazzaley A Atkins DC Heagerty PJ The use and effectiveness of mobile apps for depression: results from a fully remote clinical trial. J Med Internet Res (2016) 18(12):e330. 10.2196/jmir.6482 27998876 PMC5209607

[45] Birney AJ Gunn R Russell JK Ary DV. MoodHacker mobile web app with email for adults to self-manage mild-to-moderate depression: randomized controlled trial. JMIR Mhealth Uhealth (2016) 4(1):e8. 10.2196/mhealth.4231 26813737 PMC4748138

[46] Lau HM Smit JH Fleming TM Riper H. Serious games for mental health: are they accessible, feasible, and effective? A systematic review and meta-analysis. Front Psychiatry (2017) 7:209. 10.3389/fpsyt.2016.00209 28149281 PMC5241302

[47] Roepke AM Jaffee SR Riffle OM McGonigal J Broome R Maxwell B. Randomized controlled trial of SuperBetter, a smartphone-based/Internet-based self-help tool to reduce depressive symptoms. Games Health J (2015) 4(3):235–46. 10.1089/g4h.2014.0046 26182069

